# Malaria risk stratification in Colombia 2010 to 2019

**DOI:** 10.1371/journal.pone.0247811

**Published:** 2021-03-11

**Authors:** Julio Cesar Padilla-Rodríguez, Mario J. Olivera, Martha Liliana Ahumada-Franco, Andrea Elizabeth Paredes-Medina

**Affiliations:** 1 Ministerio de Salud y Protección Social, Bogotá, D.C., Colombia; 2 Grupo de Parasitología, Instituto Nacional de Salud, Bogotá, D.C., Colombia; 3 Grupo de Entomología, Instituto Nacional de Salud, Bogotá, D.C., Colombia; Instituto Rene Rachou, BRAZIL

## Abstract

**Background:**

Heterogeneity and focalization are the most common epidemiological characteristics of endemic countries in the Americas, where malaria transmission is moderate and low. During malaria elimination, the first step is to perform a risk stratification exercise to prioritize interventions. This study aimed to identify malaria risk strata in the ecoepidemiological regions of Colombia.

**Methods:**

This was a descriptive and retrospective study using cumulative malaria cases in 1,122 municipalities of Colombia from 2010 to 2019. To identify the strata, the criteria proposed by PAHO were adapted. To classify the receptive areas (strata 2, 3, and 4) and nonreceptive areas (stratum 1), 1,600 m above sea level, ecotypes, main malaria vector presence, *Plasmodium* species prevalence and occurrence of malaria cases were used. The area occupied by the receptive municipalities, the cumulative burden, and the at-risk population in the regions were calculated.

**Results:**

Ninety-one percent of the Colombian territory is receptive to the transmission of malaria and includes 749 municipalities with 9,734,271 (9,514,243–9,954,299) million at-risk inhabitants. Stratum 4 accounted for 96.7% of the malaria burden, and cases were concentrated primarily in the Pacific and Uraba-Bajo Cauca-Sinu-San Jorge regions. *Plasmodium vivax* predominates in most of the receptive municipalities, except in the municipalities of the Pacific region, where *P*. *falciparum* predominates. *Anopheles albimanus*, *An*. *nuneztovari* s.l., and *An*. *darlingi* were the main vectors in receptive areas.

**Conclusions:**

In Colombia, 91.2% of the territory is receptive to the transmission of malaria and is characterized by being both heterogeneous and focused. Stratum 4 contains the greatest burden of disease, with a relatively greater proportion of municipalities with a predominance of *P*. *vivax*. However, there is a low proportion of municipalities with *P*. *falciparum* mainly in the Pacific region. These findings suggest that the latter be prioritized within the malaria elimination plan in Colombia.

## Introduction

In the Americas, the major malaria transmission areas are Amazonia (Brazil, Colombia, Peru, Ecuador, Bolivarian Republic of Venezuela, Suriname, Guyana and Guyana Francesa), Mesoamerica (Guatemala, Honduras, Nicaragua, and Panama), Haiti, and the Dominican Republic [1]. In 2019, in the Americas, more than 80% of malaria cases were concentrated in the Bolivarian Republic of Venezuela, Brazil, Colombia, Guyana, Nicaragua, and Panama; however, within each country, there are areas with variable intensities [2]. Heterogeneity and focalization are the most common epidemiological characteristics of these endemic countries in the Americas, where malaria transmission is moderate and low [3,4].

Historically, to address the variability in several malaria transmission scenarios, different typologies based on epidemiology and determining factors of malaria transmission, such as clime and ecotypes, have been used to stratify malaria risks [5]. Application of these methodologies allows the establishment of smaller units or strata geographically defined according to the receptivity, which provides evidence that supports decision-making [5]. The World Health Organization (WHO) suggests malariogenic potential as a method to stratify risk malaria transmission because it is a critical factor in determining strategies to achieve elimination and prevent re-establishment of transmission [1]. Malariogenic potential is defined as the capacity of an ecosystem to favor malaria transmission (receptivity), risk of importation of the parasite (vulnerability), and vector competence (infectivity). Several methods have been proposed for assessing this potential [6,7].

In the Americas, the epidemiological stratification methodology and its application to the study of malaria distribution have been used since 1979 in endemic countries [8]. The stratification was based on different approaches; however, the majority of countries have used the trend of the annual parasite index (API) to stratify risk, which allowed the identification of strata or priority areas for malaria control [8,9]. In this region, there are several examples, such as in the state of Sucre (Venezuela), API was used to identify *P*. *vivax* transmission hotspots [10], and in Belize, Brazil and Peru, cumulative burden was used to identify areas where the highest number of cases were concentrated [11–13].

In Colombia, at the beginning of the 1980s, the API was used as a risk criterion to identify critical malaria transmission areas; additionally, this information was complemented with local determinates that contributed to the maintenance of malaria transmission [14]. Previously, it was established that more than 85% of the Colombian territory located below 1,600 m a.s.l. exhibits conditions that favor malaria transmission, and altitude is one of the most important geographical determinants that delimits malaria transmission in endemic areas. There is evidence that at 1,600 m a.s.l., the temperature is approximately 19°C (it changes approximately 0.625°C every 100 m) [15], and these conditions are suitable for the development of the sporogony cycle in malaria vector mosquitoes. Several studies have established that the minimum temperatures required for the development of *P*. *falciparum* and *P*. *vivax* in mosquitoes are approximately 18°C and 15°C, respectively, and these values delimit malaria at higher elevations [16]. Using these criteria and the ecotypes, six ecoepidemiological regions were identified that allowed elucidation of receptivity and vulnerability in all of them [17]. In most of the Colombian territory, malaria transmission exhibits endemic epidemic behavior that is persistent, variable and of moderate to low transmission [17]. *P*. *vivax* infections predominate (55.1%), and complications occur in between 1 and 2% of reported cases [18]. In Colombia 9 *Anopheles* species are considered as malaria regional and local vectors: *Anopheles (Nys*.*) darlingi*, *An*. *(Nys*.*) nuneztovari* s.l., *An*. *(Nys*.*) albimanus*, *An*. *(An*.*) calderoni*, *An*. *(Ker*.*) neivai s*.*l*., *An*. *(Ker*.*) pholidotus* (as *An*.*lepidotus*), *An*. *(An*.*) pseudopunctipennis* s.l., *An*. *(An*.*) punctimacula* s.l. and *An*. *(Nys*.*) benarrochi* B [19–22].

In Colombia, the National Strategic Plan for Malaria 2019–2022 includes strategies for malaria prevention, diagnosis, and treatment and improved surveillance and control proposed by the WHO in Global Technical Strategy for Malaria (2016–2030) to progress toward malaria transmission elimination [23,24]. The goals included in the National Strategic Plan for Malaria are to reduce malaria mortality by 80% by 2021 and morbidity by 40% by 2022 compared to 2017 [24]. The malaria stratification risk is the first step to planning prevention and control and guiding actions to prevent the re-establishment of transmission in areas where it has been interrupted. The objective of the present study was to identify malaria risk strata in the national territory using Pan American Health Organization (PAHO) methodology to generate evidence to prioritize and implement strategies that support the elimination of this disease in Colombia.

## Methods

### Study setting

Colombia is located in extreme northwestern South America with an area of 1.141.748 km^2^ and has a population of approximately 48.2 million inhabitants [25]. It is characterized by geographical diversity due to its location and the presence of the Andes Mountains that cross from south to north. These conditions and their interactions with the bioclimatic characteristics have delimited six natural regions: the Caribbean Region, located to the north and corresponding to the coastal zone on the Atlantic Ocean; Pacific Regions, in the eastern part of the country corresponding to the coastal zone on the Pacific Ocean; the Andean Region, situated in the center of this country, comprising Andes Mountain and two inter-Andean valleys; the Orinoco and Amazonia regions to the east of the Andes Mountain; and finally, an insular region composed of islands, cays and islets. Administratively, the country is divided into second-level territorial entities called departments (32) and districts (9) and third-level territorial entities called municipalities (1,122) [26].

### Study design and data sources

A descriptive and retrospective study was performed in 1,122 municipalities of Colombia using annual malaria cases from 2010 to 2019, environmental variables and, main malaria vector distributions to establish malaria risk strata according to the methodology proposed by PAHO [27].

### Data collection

Information about malaria cases was obtained from the Departmental and District Malaria Prevention and Control Programs and Integrated Information System of Social Protection (SISPRO, in its Spanish acronym; https://www.sispro.gov.co/Pages/Home.aspx). In Colombia, it is mandatory that all malaria cases reported to surveillance systems be confirmed by identification of *Plasmodium* species using microscopy diagnosis, rapid diagnostic tests or polymerase chain reaction. Microscopic examination of the blood smear is the gold standard for malaria diagnosis. Each case must be classified as indigenous or imported [28]. Records of malaria vectors from the Entomology Group of Instituto Nacional de Salud of Colombia (entomological collection database and literature search about malaria vector occurrence data registered between 2009 to 2020) and Cartographic and geographical information was obtained from the Instituto Geográfico Agustín Codazzi (SIGOT, http://www.sigotn.igac.gov.co/sigotn/). Malaria risk populations were estimated using population projections estimated from the national census conducted in 2005 obtained from the Departamento Administrativo Nacional de Estadística—DANE of Colombia (http://www.dane.gov.co/).

### Malaria risk stratification

The analysis unit for malaria risk stratification was the municipality, and to characterize the type of malaria transmission in each municipality, the ecoepidemiological region previously identified in Colombia was used [17]. Additionally, to clarify the receptivity, the ecoepidemiological regions were described using environmental variables, such as altitude, precipitation, temperature, main ecotypes, and the presence of regional and local vectors. The presence of the main malaria vectors was determined by the ecoepidemiological regions because there are no records for all municipalities, especially those with low malaria transmission or nonactive focus. All the malaria cases reported to surveillance systems were revised to confirm origin (indigenous or imported) and parasite species. Mixed infections occurring with low frequency (less than 1% of malaria cases per year) were included as *P*. *falciparum*. *P*. *malariae* cases are scarce and were not included [29].

The malaria risk population was established by municipalities, taking into account that in Colombia, malaria transmission is primarily rural. All inhabitants estimated in projections of DANE in rural areas in municipalities located below 1,600 m a.s.l. were included; additionally, municipalities located in Pacific and Amazonas regions included urban populations.

Using the methodology proposed in the Manual of Stratification according to the Risk of Malaria and Elimination of Transmission Focuses—Americas Region [27], municipalities were classified according to receptivity (the ability of the ecosystem to allow the transmission of malaria) and vulnerability (the probability that malaria parasites will be imported). To identify the receptivity of municipalities, 1,600 m a. s. l. was used as a cutoff point, and this altitude was established as the maximum altitude that favors development of *Plasmodium species* in the malaria vectors and the environmental conditions for malaria transmission. Nonreceptive municipalities were those located above this altitude.

To assign a stratum to each municipality, PAHO recommendations for the classification of malaria risk strata were adapted according to the available information in malaria surveillance systems of Colombia, and the exercise was realized step by step, starting from the classification of stratum 1 (S1), followed by stratum 4 (S4, active foci) and stratum 3 (S3, nonactive and residual foci). Finally, the remaining municipalities were included in stratum 2 (receptive only):

**Stratum 1 (S1):** nonreceptive municipalities located above 1,600 m above sea level in which there is no proven risk of malaria transmission.
**Stratum 2 (S2):** receptive but not vulnerable municipalities without indigenous or imported cases from endemic areas or border endemic countries.
**Stratum 3 (S3):** receptive and vulnerable municipalities without indigenous cases in the last four years or residual foci with indigenous cases (≤ 200 cases per year) for at least five years in the last decade.
**Stratum 4 (S4):** receptive municipalities with indigenous cases from active foci.

### Data analysis

All data were stored in a standard format in MS Excel (Microsoft, Redmond, USA) and were analyzed using Stata (release 15, Stata Corporation, College Station, TX, USA), while ArcGIS version 10.5 (ESRI, Redlands, CA) was used to produce maps. Each municipality categorized in the malaria transmission risk strata was verified to meet the criteria for each stratum.

Summary statistics were constructed for the entire dataset by developing absolute frequency measures, such as accumulated cases by region and municipality, territorial extensions (in km^2^) and the number of inhabitants at risk (confidence interval of projection). To establish the absolute risk of transmission by regions and municipalities in S4, relative frequency measures were constructed as percentages, the proportion of *Plasmodium falciparum* (PPf), and the median annual parasitic index (APIm) per 1,000 inhabitants in the study period. Classification of the intensity of transmission in S4 was performed from percentile (25th-75th) as follows: very high intensity substratum: APIm 148.1–38.5 x 1,000 inhabitants; high intensity substratum: APIm: 38.4–14.7 x 1000 inhabitants; medium intensity substratum: APIm 14.6–6.1 x 1000 inhabitants and low intensity substratum: APIm 6.0–0.1 x 1,000 inhabitants. Correlations between APIm and cumulative malaria cases were observed, and maps were developed separately for the strata, ecoepidemiological regions, and *Anopheles* species distributions.

### Ethics statement

The present study met the ethical requirements established in Resolution 8430 of 1993 of the Ministry of Health of Colombia, Article 11, which establishes that studies, such as the present one, are risk-free and do not require approval by the Ethics Committee. Confidentiality and anonymity of the data were guaranteed.

## Results

### Ecoepidemiological region descriptions

In Colombia, malaria transmission primarily occurs between 0 and 1,600 m a.s.l. where the temperature fluctuates between 17 and 34°C and the relative humidity is not greater than 90%. From the natural regions of Colombia, the ecoepidemiological regions of malaria transmission were defined, however it was necessary to define the Uraba-Bajo Cauca-Sinu-San Jorge region considering that the transmission dynamics is different from the natural regions that surround it. The most intense transmission occurs in the Pacific and Uraba-Bajo Cauca-Sinu-San Jorge, where transmission occurs in ecotypes, such as mangroves, floodplains, tropical rainforests, and savannas (Table 1).

**Table 1:** Description of ecoepidemiological regions for malaria transmission in Colombia.

Ecoepidemiological region	Environmental variables [15,30]				Climate classification Köppen-Geiger [31]	Ecotypes [32]	Main regional and local vector** [19,20,22,33–37]
	Altitude range (m a.s.l.)	Precipitation (mm/year)	Temperature (°C)	Relative humidity (%)			
Pacific	0–1,100	3,000–9,000	18–30	89	Equatorial rainforest Equatorial monsoon	Mangroves Coastal rainforest High rainforest Floodplains	*An*. *albimanus* *An*. *darlingi* *An*. *nuneztovari* s.l. *An*. *neivai* s.l. *An*. *calderoni*
Uraba-Bajo Cauca-Sinú-San Jorge	0–1,600	800–3,600	18–30	85	Equatorial monsoon Equatorial savannah Equatorial rainforest	Flood plains Savannas Rainforest	*An*. *nuneztovari* s.l. *An*. *albimanus* *An*. *darlingi*
Amazonia	80–400	3,000–4,500	17–32	85	Equatorial rainforest	Rainforest Flood plains Savannas	*An*. *darlingi* *An*. *benarrochi* B
Orinoco	80–500	1,500–3,500	19–34	82	Equatorial savannah Equatorial monsoon	Piedmont Flood plains Savannas Gallery forest	*An*. *nuneztovari* s.l. *An*. *darlingi* *An*. *albitarsis* s.l.
Caribbean***	0–865	500–2,000	20–34	80	Equatorial savannah Desert climate	Mangroves Tropical dry forest	*An*. *albimanus* *An*. *darlingi*
Andean (Only Tropical region)*	100–1,100	800–5,000	18–34	82	Equatorial savannah Equatorial monsoon Equatorial rainforest	Foothill Rainforest	*An*. *nuneztovari* s.l. *An*. *darlingi* *An*. *albimanus*

* Total Andean region 100 ≥ 4,100 m a.s.l.

** Natural infectivity with *Plasmodium* spp. in the last 15 years.

***Municipalities of the insular region were included in the Caribbean region.

### Receptive and nonreceptive areas

**Fig 1:**
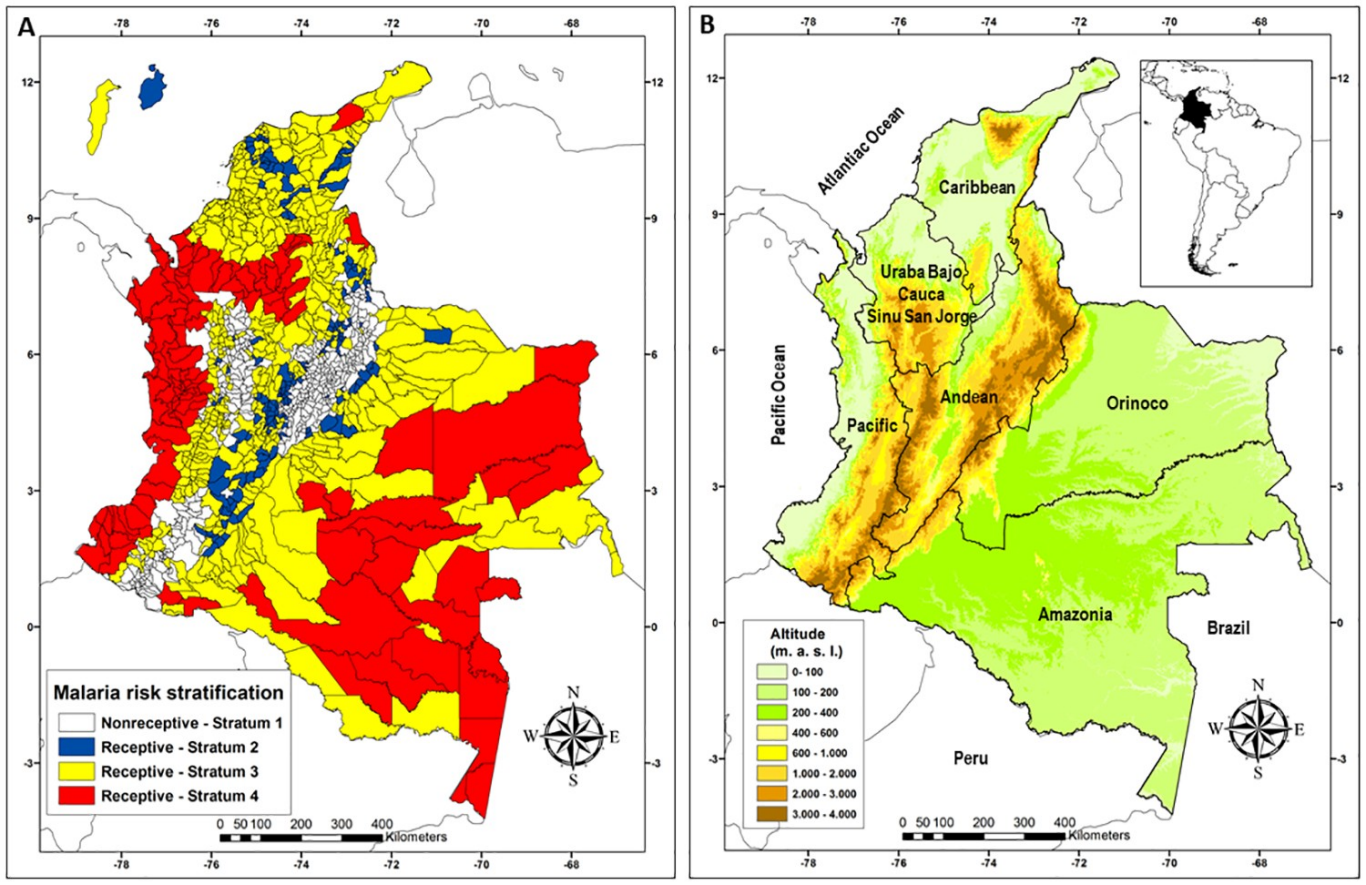
Risk strata of malaria transmission and ecoepidemiological regions in Colombia, 2010 to 2019. **A**) Malaria Risk Strata and **B)** Eco-epidemiological regions and topography.

Receptive areas (S4, S3, and S2) with and without indigenous transmission of malaria included 749 municipalities, which accounted for 66.6% of the municipalities in the country (749/1,122). This area encompasses 1,043,003 km^2^, representing 91.2% of the national territory (Fig 1, Table 2). The at-risk population was approximately 9,734,271 (9,514,243–9,954,299) inhabitants (Table 3), and the cumulative malaria burden between 2010 and 2019 was 607,042 cases (Table 3). Strata with and without indigenous transmission but at risk of importation of the parasite (vulnerable), i.e., S4 and S3, respectively, had a territorial area of 990,712 km^2^, representing 86.7% of the national territory (Table 2). During the study period, 607,042 cases were reported in these strata that involved 583 municipalities (Table 3).

**Table 2:** Municipalities and area by malaria risk stratum and ecoepidemiological regions in Colombia.

Region	S4		S3		S2		S1	
	Number of municipalities	Area (km^2^)	Number of municipalities	Area (km^2^)	Number of municipalities	Area (km^2^)	Number of municipalities	Area (km^2^)
Pacific	43	78,226	66	26,547	0	0	70	26,782
Uraba-Bajo Cauca Sinu San Jorge	27	38,757	75	32,100	6	1,063	47	16,071
Amazonia	20	244,375	35	161,076	0	0	4	1,082
Orinoco	7	125,767	45	123,598	5	3,872	2	495
Caribbean	8	12,062	121	80,138	38	14,721	0	0
Andean	2	3,601	134	64,464	117	32,636	250	55,787
	**107**	**502,788**	**476**	**487,924**	**166**	**52,291**	**373**	**100,217**

**Table 3:** At-risk population and cumulative burden of malaria by stratum and ecoepidemiological region in Colombia from 2010 to 2019.

Region	At-risk population			Cumulative cases		
	S4	S3	S2	S4	S3	S2*
Pacific	1,330,464	448,013	0	319,425	1,395	0
Uraba-Bajo Cauca Sinu San Jorge	657,453	1,098,113	27,092	184,760	7,072	0
Amazonia	373,887	638,079	0	44,682	1,837	0
Orinoco	113,523	1,038,168	15,514	8,357	2,155	0
Caribbean	113,347	1,609,216	178,665	18,804	4,481	0
Andean	46,170	1,389,198	657,369	10,728	3,346	0
Total	**2,634,844**	**6,220,787**	**878,640**	**586,756**	**20,286**	**0**

* Burden malaria cases S2 = 0 in all regions.

In Colombia, areas with indigenous transmission, S4, had a territorial area of 502,788 km2, which was 48.2% of the receptive area, including 107 municipalities (14.3% municipalities of the receptive area) (Table 2) and 2,634,844 (2,575,287–2,694,401) at-risk inhabitants (Table 3). In S4, the cumulative burden of disease between 2010 and 2019 was 586,756 cases, representing 96.7% of the cases registered in the country (Table 3).

**Fig 2:**
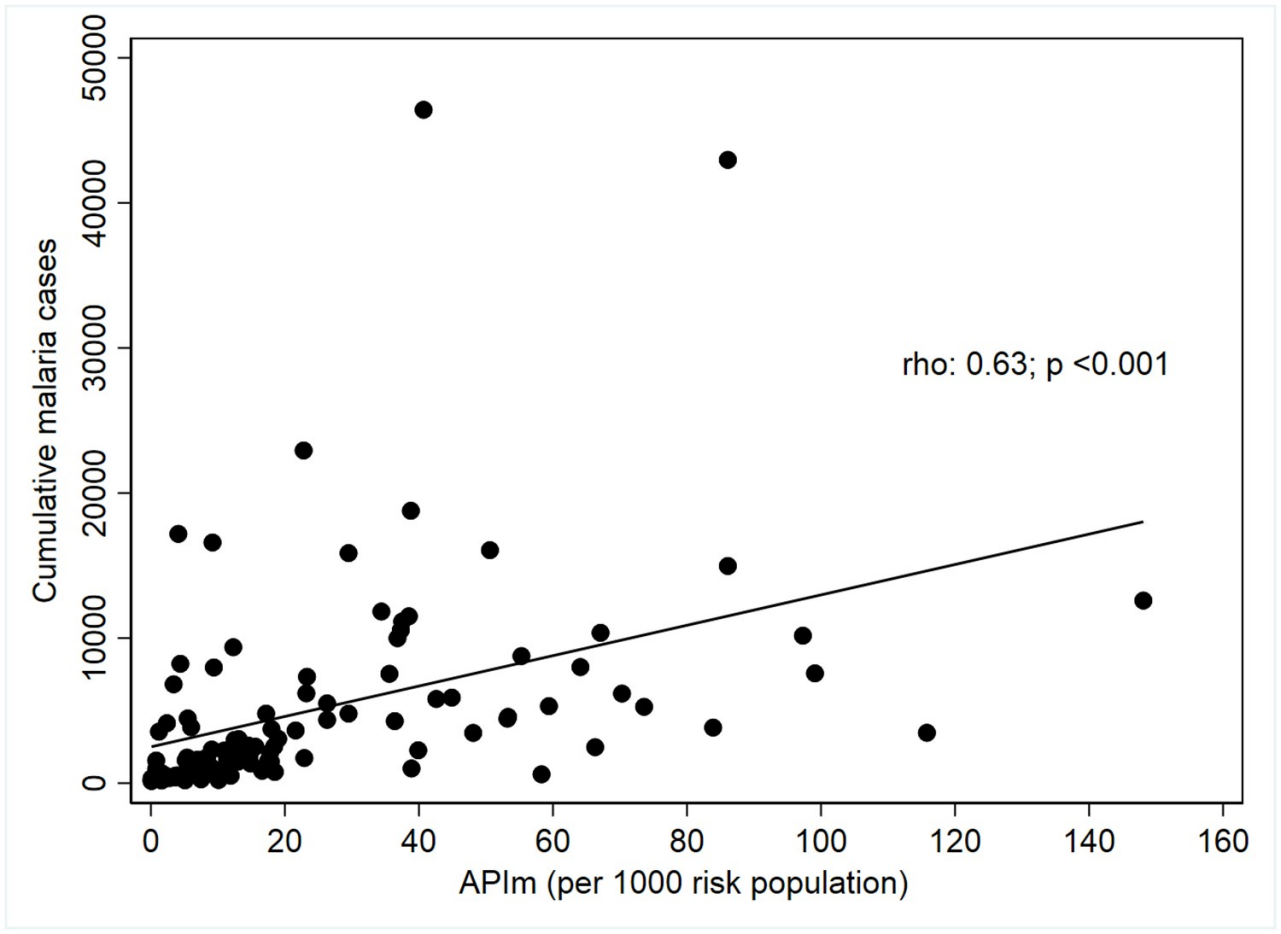
Univariate correlations of the median annual parasite index (APIm) per 1000 inhabitants with cumulative malaria cases from 2010 to 2019 in Colombia.

Using variables on the risk and intensity of malaria transmission, S4 was characterized, and the results provide evidence showing the heterogeneity in the municipalities by ecoepidemiological regions. The Uraba-Bajo Cauca-Sinu San Jorge and Pacific regions contribute 92.2% of malaria cases registered in the country, and the absolute risk was 23.2 and 22.4 per 1,000 inhabitants, respectively (Table 4). Furthermore, it was established that in Colombia, there is a statistically significant, moderate, and directly proportional linear relationship between the accumulative burden of malaria cases and the APIm (Rho = 0.63; p <0.001) (Fig 2).

**Table 4:** Cumulative cases, annual parasite index median (APIm), proportion of *Plasmodium falciparum* infections (FRIF), and malaria transmission intensity in S4, Colombia from 2010 to 2019.

Region	Epidemiological variables					Proportion of municipalities by malaria transmission intensity*				
	Cumulative cases 2010–2019	Proportion cases by country	% variation 2010–2019**	APIm X 1,000 Inh.	PPf (%)	Number of municipalities	% Very high	% High	% Median	% Low
Pacific	550,450	53.7	35 (+)	22.4	69	43	33 (14/43)	42 (18/43)	18 (8/43)	7 (3/43)
Uraba-Bajo Cauca Sinu San Jorge	393,956	38.5	76 (-)	23.2	18	29	17 (5/29)	28 (8/29)	24 (7/29)	31 (9/29)
Amazonia	48,577	4.7	20 (-)	14.7	16	20	20 (4/20)	5 (1/20)	35 (7/20)	40 (8/20)
Orinoco	4,462	0.4	18 (-)	6.7	17	5	0	0	20 (1/5)	80 (4/5)
Caribbean	16,283	1.6	63 (-)	16.0	19	8	25 (2/8)	0	50 (4/8)	25 (2/8)
Andean	10,713	1.0	72 (+)	20.8	3.3	2	50 (1/2)	0	0	50 (1/2)
**Total**	**1,024,441**	**100.0**	**34.2 (-)**	**17.8**	**43**	**107**	**24. 1 (26/107)**	**25.3 (27/107)**	**25.3 (27/107)**	**25.3 (27/107)**

*Very high: APIm 148,1–38,5; high: APIm 38,4–14,7; Median: APIm 14,6–6,1; Low: APIm 6,0–0,1.

**(+) increase, (-) decrease.

In the Pacific region, a PPf of 69% was registered, in contrast to the values of the other regions, which did not exceed 19%, indicating a higher *P*. *vivax* infection prevalence. During the study, it was observed that four regions decreased the cumulative malaria cases by 18–76%, but in the Pacific and Andean regions, the increases were 35% and 72%, respectively (Table 4).

Regarding the malaria transmission intensity in the municipalities for each of the ecoepidemiological regions, it was observed that in the Pacific region, 75% belonged to very high and high categories, whereas in the Uraba Bajo Cauca Sinu San Jorge, 45% were between these categories, and 55% were municipalities with median or low intensity. In the Andean region, only two municipalities were classified in S4, one with high intensity and the other with low intensity (Table 4).

**Fig 3:**
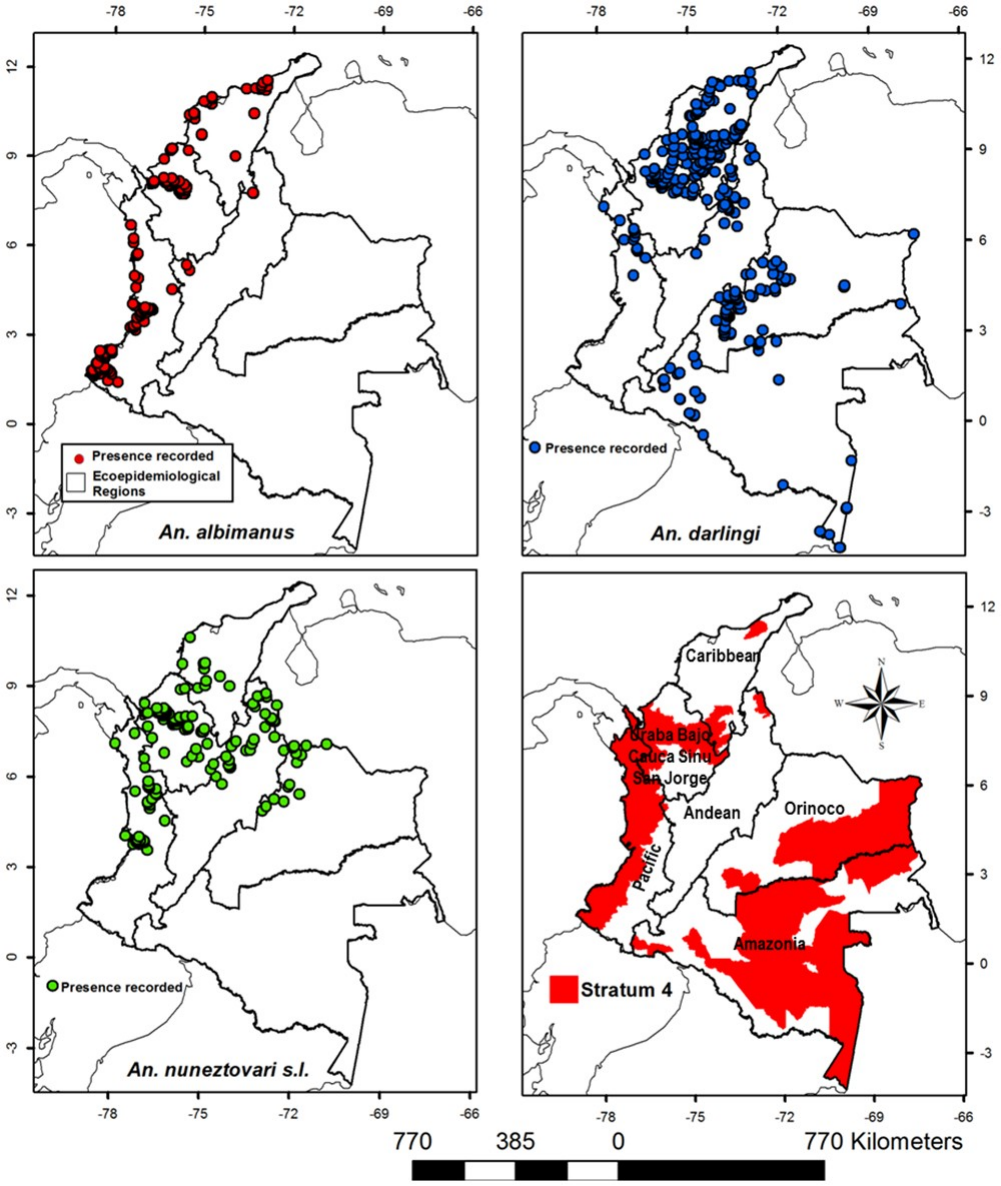
Distribution of *Anopheles albimanus*, *An*. *darlingi*, and *An*. *nuneztovari* s.l., the primary malaria vectors in Colombia by ecoepidemiological region and stratum 4.

In S4, *An*. *nuneztovari* s.l., *An*. *darlingi* and *An*. *albimanus*, the primary malaria vectors in Colombia, were present. In the Uraba-Bajo Cauca-Sinu-San Jorge region, 46,000 km^2^ was a suitable habitat for *An*. *nuneztovari* s.l., 36,150 km^2^ was a suitable habitat for *An*. *darlingi*, and 35,200 km^2^ was suitable habitat for *An*. *albimanus*, indicating that over 80% of this region area had suitable environmental conditions for these species to sustain malaria transmission. In the Pacific region, the distribution of *An*. *albimanus* (55,660 km^2^), *An*. *nuneztovari* s.l., (28,560 km^2^), and *An*. *darlingi* (14,500 km^2^), showed that 80% of this region had habitat suitability to allow at least one of the primary vectors to be present. In S4 of the Orinoco and Amazonian regions, the main malaria vector was *An*. *darlingi* (Fig 3).

S3 contained 476 municipalities (42.4%) with a territorial area of 487,924 km^2^ (46.8% of the receptive area) (Table 2) and an at-risk population of 6,220,787 (6,080,176–6,361,398) inhabitants (Table 3). During the study period, 20,286 imported malaria cases were reported in this stratum. In contrast, S2, which includes 166 receptive municipalities without a risk of the importation of parasites, had a territorial area of 52,292 km^2^ (Table 2) and an at-risk population of 878,640 (858,780–898,500) inhabitants (Table 3), which was the smallest of the strata. Finally, the nonreceptive area (S1) was located at an altitude greater than 1,600 m a.s.l. This area contained 373 municipalities (33.2%) and had a territorial area of 100,217 km^2^ (Table 2).

### Strata distribution by ecoepidemiological regions

Pacific and Uraba-Bajo Cauca-Sinu-San Jorge ecoepidemiological regions accounted for 84.5% of the cases (512,652) registered in Colombia between 2010 and 2019. The major cumulative burden of malaria of these regions was registered in the receptive area with indigenous transmission (S4) and with 504,185 cases (98,3%) compared to the 8,467 cases (1,7%) in the receptive and vulnerable areas (S3) (Table 3). Malaria transmission was concentrated in 72 municipalities, 43 in the Pacific region, and 29 in the Uraba-Bajo Cauca-Sinu-San Jorge region (Table 2). The remaining 15.5% of the cases registered in the country were distributed in the other five ecoepidemiological regions: Amazonia, 7.7%, Caribbean, 3.8%, Andean, 2.3%, and Orinoco, 1.7% (Table 3).

In the Pacific region, S4 had an area of 78,226 km^2^ and an at-risk population of 1,330,464 (1,300,391–1,360,537) inhabitants, corresponding to a population density of 17 inhabitants per km^2^, distributed in 43 endemic municipalities (Tables 2 and 3). In the Uraba-Bajo Cauca-Sinu-San Jorge region, S4 had an area of 38,757 km^2^ and an at-risk population of 657,453 (642,592–672,314) inhabitants, corresponding to a population density of 16.7 inhabitants per km^2^ distributed in 29 endemic municipalities (Tables 2 and 3).

S3 consisted of 476 municipalities distributed in all ecoepidemiological regions and represented 42,7% of the national territory. This indicated that Colombia has a large receptive area with high vulnerability throughout the country (Table 2, Fig 1). Amazonas, Orinoco, and the Caribbean were the regions that most contributed to this stratum, with 364,812 km^2^ or 74.8% of their area (Table 2). S2 extended throughout 4.6% of the Colombian territory and was distributed among different ecoepidemiological malaria transmission regions; however, the Andean and Caribbean regions accounted for 90.6% of the total area classified as S2 (Table 2, Fig 1).

## Discussion

In this study, four strata were established for the risk of malaria transmission in Colombia, confirming that there are areas where a greater burden of the disease is concentrated. This stratification exercise demonstrated that more than 90% of the Colombian territory has receptivity and vulnerability conditions that favor the maintenance of malaria transmission (1,043,003 km2). This territory includes areas of active and heterogeneous transmission with variable intensity. Despite the large extension, the greatest burden of the disease was concentrated in 107 (14%) municipalities of the receptive area, and the remaining 86% (642 municipalities) currently register low transmission (≤ 200 cases per year) or do not register autochthonous cases. However, they present a high vulnerability given that they present environmental conditions and the presence of vector malaria that would favor the occurrence of cases as a consequence of the importation of cases from sources of active transmission. Additionally, the use of the two approaches to characterize transmission in S4, burden malaria cases and API made it possible to identify the municipalities with a higher burden of the disease within the epidemiological regions, and with the API, the absolute risk was established in each ecoregion. The two approaches are complementary because they allow evidence of heterogeneity within regions and between regions. This malaria transmission pattern has been observed in regions with moderate to low transmission intensities in the Americas, as described in the state of Bolívar (Venezuela) and the Amazonian region (Brazil) [38–40].

In Colombia, the receptive areas S4, S3, and S2 are predominantly rural, and they are continually exposed to environmental changes caused by human influences. These changes are associated principally with deforestation, intensification of mining activities, and illicit crops that have contributed substantially to modifying the distribution and incidence of malaria [29,41,42]. Additionally, the high mobility of the population, immigration from endemic border countries, susceptibility of the human population to infection, and access to health services favor an increase in vulnerability in several risk strata of malaria transmission, especially S3, which is receptive and has a high risk of importing parasites from endemic areas [3,5,17].

Another important characteristic of the receptive area is the presence of several ecotypes where there is high mobility of the population and increased vulnerability, which explains the diversity of the epidemiological pattern of malaria transmission in Colombia. These epidemiological patterns can be explained using the ecoregional approach proposed by Rubio-Palis and Zimmerman [43], who identified five ecoregions using malaria vector distributions and environmental variables: coastal, piedmont, savanna, interior lowland forest and high valley [5,17,43,44].

The malaria risk stratification showed that S4 (presence of indigenous cases) occurs on the coast, floodplains, alluvium, rainforest and in the western Andean piedmont in the Pacific regions and savannas, valleys and floodplains in the Uraba-Bajo Cauca-Sinu-San Jorge region. The main malaria vectors, *An*. *albimanus*, *An*. *nuneztovari* s.l. and *An*. *darlingi*, can be sympatric in many of these regions [20,33,34,45,46]; however, the ecotype diversity in S4 favors the occurrence of local vectors such as An. *neivai* s.l. along the coast of the Pacific region [35,47], and *An*. *calderoni* in the southwestern area of the same region (coastal ecoregion) [22,33,37], and this diversity contributes to sustaining malaria transmission. In the Orinoco and Amazonian regions, S4 corresponds to the interior lowland forest ecoregion, savannas, floodplain and, rainforest and the main malaria vector is *An*. *darlingi* [19,48,49]. Furthermore, recent studies have identified *An*. *benarrochi* B as a local malaria vector in S4 in southwestern Amazonia (Andean Piedmont, Putumayo department) [21].

S3 (receptive and vulnerable areas) is present in coastal, piedmont, savanna, and interior lowland forest ecoregions [43]. The coastal ecoregions contain the Caribbean and the northern part of the Uraba-Bajo Cauca-Alto San Jorge-Sinu ecoepidemiological regions, and in these areas, *An*. *albimanus* and *An*. *darlingi* are the main vectors that contribute to malaria transmission [19,22,34]. The Piedmont ecoregion includes Eastern Andean municipalities located in the lowlands of the Inter-Andean valleys where the main malaria vector is *An*. *nuneztovari* s.l. [50,51], although *An*. *pholidotus* has also been incriminated in the southeast of this region as a local malaria vector [52]. On the other hand, S3 is found in the Orinoco and Amazonian regions, which include savanna, rainforest and interior lowland forest ecoregions. In the savanna, located north of the Orinoco region, the main vector is *An*. *nuneztovari* s.l., and *An*. *darlingi* is the main malaria vector in the remaining municipalities of these regions [19,36,53].

The stratification and description of S4 showed that there are few municipalities with a predominance of *P*. *falciparum* transmission that are located in the Pacific region. This is an opportunity to prioritize these areas to implement strategies to start eliminating transmission of this parasite in Colombia [54]. On the other hand, municipalities with *P*. *vivax* transmission are distributed throughout ecoepidemiological regions, and the elimination of this parasite species represents a challenge to malaria elimination due to the presence of mature gametocytes at an early stage of infection, resulting in greater transmissibility and relapse by the activation of dormant hypnozoites [55–58].

The major limitation of this study was information bias in the data and secondary sources.

In the surveillance system, only cases that come to official health services for a diagnosis of malaria are reported, and there is little information on the entities that perform diagnoses outside the system, which could lead to under registration. Furthermore, it is likely that in some registries, there is a misclassification of indigenous and imported cases. To control this bias, the data were cleaned to improve analysis efficiency and ensure correct municipality strata classification according to the criteria defined in the study. Due to available information on malaria cases by municipalities, it was necessary to adjust the strata criteria, especially for S3, because the information did not allow us to clearly define the conditions of active and nonactive residual foci when the municipalities had a low burden of disease. Another limitation was that malaria vector information to confirm receptivity in low malaria transmission municipalities or currently residual nonactive foci was scarce.

This study is the first malaria risk stratification exercise in Colombia that follows the methodology proposed by PAHO adjusted to the current situation of malaria transmission in the country [27]. Although it was only possible to obtain general information up to down to third-level administrative division (municipalities), this allowed us to classify all municipalities in their corresponding strata and select priority municipalities that should be included in the strategic plans for the elimination of malaria. In these municipalities, it is necessary to continue conducting microstratification exercises for the design and implementation of sustainable operational plans to provide solid evidence for the appropriate selection of specific, cost-effective, and sustainable interventions for developing plans to eliminate and prevent the re-establishment of malaria transmission in Colombia. In these areas there is also the presence of other prevalent infectious diseases [59], for which health policies must be implemented that address as many of the social determinants of health as possible in order to contain social costs [60]. Knowledge of the different potential patterns of transmission of the disease in the strata with a predominance of *P*. *falciparum* facilitates the definition of the specific goals, objectives and targets according to the priorities established in the elimination plans. However, continuously updating the malaria risk strata is required due to the presence of municipalities where it is mandatory to regularly characterize the transmission dynamics to confirm the presence of nonactive residual foci and register imported cases to implement surveillance actions and prevent reintroduction.

## Conclusions

Ninety-one percent of the Colombian territory is receptive and vulnerable to malaria transmission. The transmission risk was heterogeneous and classified into four strata adapted to local transmission dynamics from indigenous transmission strata to nonreceptive strata without indigenous cases and no vulnerability. It was also established that the transmission of *P*. *falciparum* mainly occurs in Pacific regions, and these areas could be considered a starting point to implement malaria elimination plans in Colombia.
